# Convective meniscus splitting of polysaccharide microparticles on various surfaces

**DOI:** 10.1038/s41598-020-80779-z

**Published:** 2021-01-12

**Authors:** Kosuke Okeyoshi, Miki Yamashita, Kulisara Budpud, Gargi Joshi, Tatsuo Kaneko

**Affiliations:** 1 Energy and Environment Area, Graduate School of Advanced Science and Technology, Japan Advanced Institute of Science and Technology, 1-1 Asahidai, Nomi, Ishikawa 923-1292 Japan; 2 Present Address: B CUBE, Center for Molecular and Cellular Bioengineering, Technische Universität Dresden, Tatzberg 41, 01307 Dresden, Germany

**Keywords:** Soft materials, Colloids, Fluids, Gels and hydrogels, Liquid crystals, Polymers, Self-assembly, Wetting, Bioinspired materials, Surfaces, interfaces and thin films

## Abstract

In contrast to convective self-assembly methods for colloidal crystals etc., “convective meniscus splitting method” was developed to fabricate three-dimensionally ordered polymeric structures. By controlling the geometry of evaporative interface of polymer solution, a deposited membrane with uniaxial orientation and layered structures can be prepared. Here it is demonstrated that xanthan gum polysaccharide microparticles with diameter ~ 1 µm can bridge a millimeter-scale gap to form such a membrane because the capillary force among the particles is more dominant than the gravitational force on the evaporative interface. This method is applicable for various substrates with a wide range of wettability (water contact angle, 11°–111°), such as glass, metals, and plastics. The specific deposition can be also confirmed between frosted glasses, functional-molecules-modified glasses, and gold-sputtered substrates. By using such a universal method, the membrane formed on a polydimethylsiloxane surface using this method will provide a new strategy to design a functional polysaccharide wall in microfluidic devices, such as mass-separators.

## Introduction

Many strategies have been explored to fabricate colloidal crystals from microparticle dispersions based on convective self-assembly techniques developed using polymeric microspheres on planar solid substrates^1–8^. However, because one side of the particle is connected to or fixed on the substrates in these methods, the flexibility of the polymers is limited, restricting the use of both sides in separation membranes and stretchable materials^9,10^. Recently, we reported the unique phenomenon of meniscus splitting, with characteristic polymeric deposition on an evaporative air–liquid interface^11^. By drying an aqueous polymer solution from a cell with a 1-mm gap, the polymer deposits at specific positions with ~ 10 mm intervals in the cell width direction by bridging the gap to form membranes in the depth direction. The process is strongly related to viscous fingering patterns; splitting occurs by solidification via the drying of a polymer solution^12–18^.

**Figure 1:**
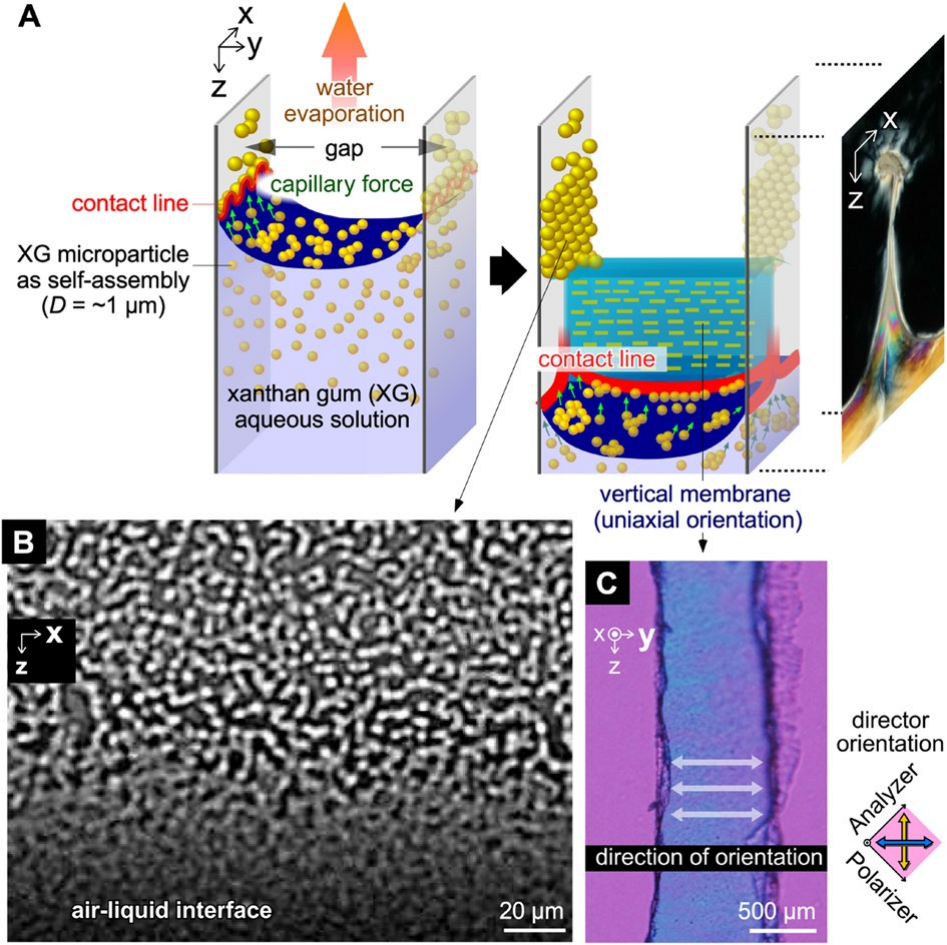
Deposition of polysaccharide microparticles from evaporative air–LC interface. (**A**) Schematic of microparticle depositions on evaporative air–LC interface and vertical membrane formation. (**B**) Optical microscopic images of XG deposited on a substrate. Initial concentration of XG: 0.5 wt%. Drying at 60 °C. (**C**) Microscopic image of dried vertical membrane through crossed Nicols with a first-order retardation plate (λ = 530 nm). Inner dimensions of the cell: (X-width, Y-thickness, Z-depth) = (15 mm, 1 mm, ~ 20 mm).

Here, we have developed a novel technique of convective meniscus splitting. Unlike the typical convective self-assembly technique, in this case, deposition occurs not only on solid substrates, but also between two air phases to form a membrane having a thickness of several tens of micrometers. Therefore, the polymeric membrane bridged the gap does not have different two sides because both sides are prepared from evaporative air–liquid interface. This technique appears applicable to various types of viscous polymer solutions, among which liquid crystalline (LC) polysaccharides were chosen to orient the self-assembled structures on the evaporative interface^19^. The technique has been demonstrated using several LC polysaccharides having megamolecular weights, such as sacran (> 10^7^ g/mol) and xanthan gum (XG; > 10^6^ g/mol)^20–22^. XG can self-assemble as ~ 1-µm-diameter microparticles dispersed stably in an aqueous solution. By drying the aqueous dispersion of XG over a millimeter-scale gap, a membrane bridging the gap is deposited having a three-dimensionally ordered structure (Fig. 1A). The distance of the cell gap strongly affects on the membrane formation and the phenomena occurs typically less than 1 mm^20^. This should be because of the capillary length, ~ 2 mm which is an effective distance for capillary force from a plane substrate^23^. Furthermore, such a membrane with crosslinking points could behave as a uniaxially swellable hydrogel. These factors indicate that such polymer membranes can be assembled on many types of substrates with suitably modified preparation conditions.

In this study, meniscus splitting is examined by controlling the direction of the gravitational force and the substrate surface wettability. In our previous reports^11,19–22^, the demonstrated conditions for the meniscus splitting^11^ were limited: the water evaporation direction was parallel to the direction of the gravitational forces, and the space was sandwiched in between the non-modified smooth surface of glass substrate. However, for the development of the splitting method, it is important to clarify and expand the possibility of the conditions. Differing from previous reports^11,19–22^, the phenomena is demonstrated under various effects from outer environments such as the gravitational forces and the substrate surface wettability to develop the phenomena into a fixed method. Actually, meniscus splitting occurs under many conditions, i.e., it is ultimately independent of gravitational forces and substrate wettability; therefore, ordered membranes can be designed under various conditions. Near the evaporative interface, the capillary force typically acts within the effective distance from a plane substrate; it is dominant for liquids between two walls separated by a 1-mm gap rather than the gravitational force^23^. Furthermore, meniscus splitting is tested on various substrate materials, such as glass with/without functional-molecules-modification, metals, and plastics with a wide range of wettability, by checking the initial adsorption on these substrates.

## Results and discussion

The viscous solution of XG was poured into a one-side-open cell of 15 mm × 1 mm ×  ~ 20 mm at ~ 25 °C. The 1.5-wt% aqueous solution has extremely high viscosity (~ 0.4 Pa s at 25 °C and ~ 0.6 Pa s at 40 °C at shear rate 10 s^−1^)^22^. The sample was placed under atmospheric pressure in an oven with an air circulator and the humidity was maintained at 24 ± 5% relative humidity (RH) at 40 °C as measured by a hygrometer. This value is theoretically supported by an estimation from the Standard Mollier diagram in the range 13–35% RH (SI Fig. S2). By focusing on the deposited polymer, the self-assembled microparticles integrated on the substrate (Fig. 1B and Supplementary Information Fig. S1) and bridge the 1-mm-gap to form vertical membrane. The dried membrane was peeled off from the cell by mechanical widening of the cell gap and mechanical cutting from the substrates. From polarized microscopic observation with a first-order retardation plate (λ = 530 nm), the membrane showed significant blue, indicating the polymer orientation in the gap direction (*Y*-direction) (Fig. 1C, and Fig. S3). From images observed by scanning electron microscope, the microparticles with diameters of 1–2 µm could be observed on the surface.

**Figure 2:**
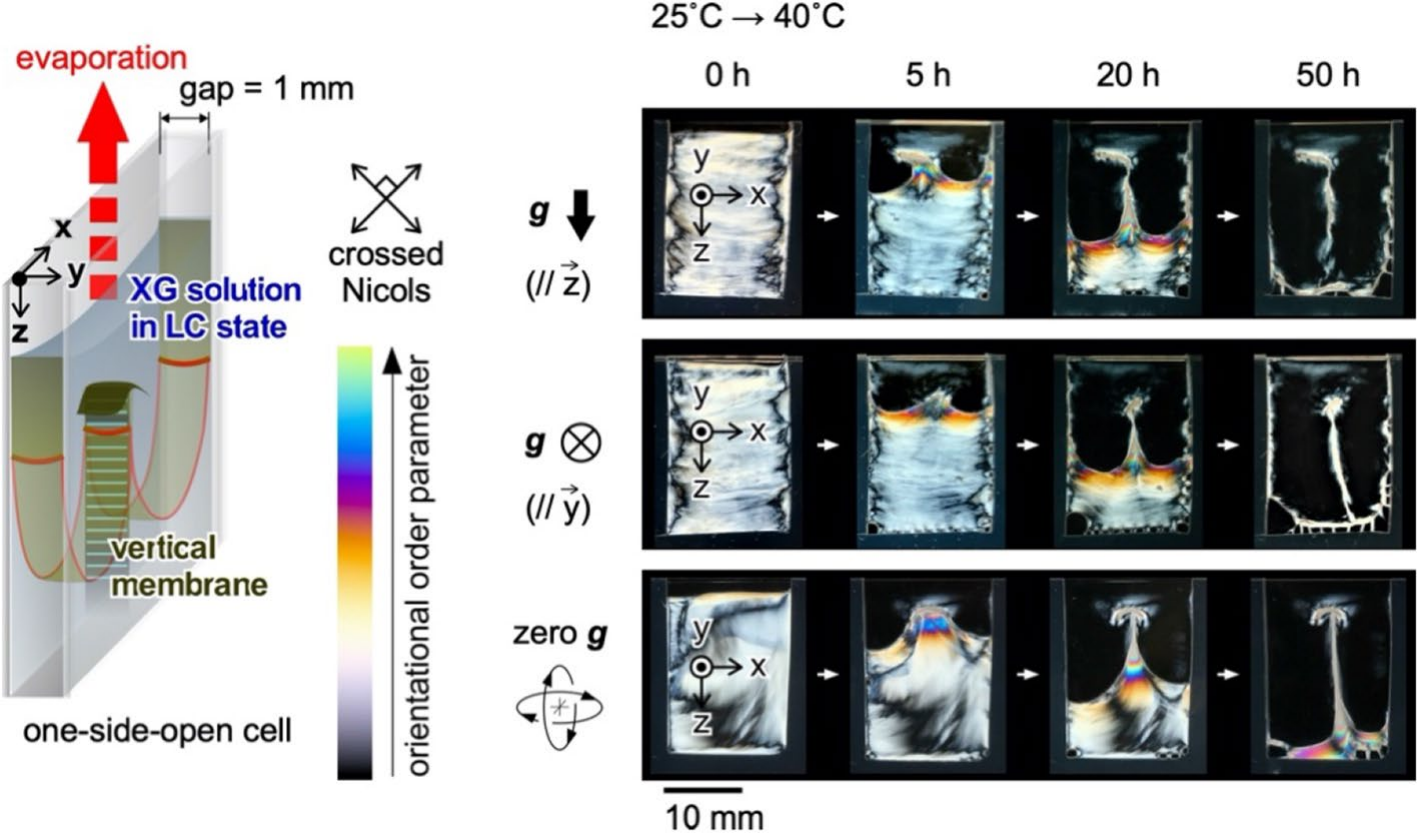
Effect of gravity on meniscus splitting and vertical membrane formation. Schematic of drying polymer LC solution from one-side-open cell and photographs of deposited polymer under cross-polarized light under conditions such that the gravitation force is parallel to the Z-axis, the Y-axis, and zero-gravity. Inner dimensions of the cell: (X-width, Y-thickness, Z-depth) = (15 mm, 1 mm, ~ 20 mm). Initial polymer concentration: 1.5 wt%. Drying temperature: 40 °C.

As shown in Fig. 2 and Fig. S4, the effect of gravity on the specific deposition is investigated by comparing three cases with controlled evaporation directions, using gravity parallel to the *Z*-axis, parallel to the *Y*-axis, and equal to zero. Under these conditions, the viscous solutions are retained in the cell without outflowing during drying. The XG deposition is monitored during water evaporation via crossed Nicols observation from the side to evaluate liquid crystallinity^24,25^. In the conditions at 25 °C and 40 °C (Fig. 2), the polarized images of the XG solution showed strong light transmission, indicating that the liquid crystallinity. The cross-polarized images also show slight changes in the bulk during drying, implying the low mobility of XG microparticles in the cell. Similar meniscus splitting and specific deposition are observed when the direction of gravitational force is vertical or arbitrary relative to the direction of evaporation from the cell. Thus, the effect of gravity on meniscus splitting is negligible during evaporation from the 1-mm gap. Actually, the thickness of the vertical membrane in the *X*-direction was measured by a micrometer, and it was in the range of 10–30 µm in all cases.

Considering that the gravitational force is small, the dominant forces for this splitting should be the viscous force and the inertia force. From a view point of fluid dynamics, the initial state of the phenomena is related on plane Poiseuille flow and Reynolds number as following equations:

1 $$U\left(y\right)=\frac{1}{2\rho \nu }\frac{dp}{dz}(y-\Delta {y}_{0})y$$

2 $$Re\equiv \frac{UL}{\nu }$$

(*U*(*y*) is the flow velocity, *ρ* is the fluid density, *ν* is the kinematic viscosity, *dp/dz* is the pressure gradient, *L* is the characteristic length). Because that the *U*, *ρ*, and *ν* are strongly dependent on time in the splitting phenomena accompanying polymer deposition, the equation should be extended by adding the spatio-temporal factors. At least, the *Re* in the initial state is extremely small, suggesting that the viscous force is extremely larger than the inertia force: e.g., the *Re* value is estimated to be 1.09 × 10^–5^ at 40 °C for 1.5 wt% solution (*ν* = *µ*/*ρ* = 0.56 m^2^ s^−1^, *U* = 1.7 mm h^−1^, *L* =  ~ 1 µm), and 1.01 × 10^–5^ at 60 °C for 1.0 wt% solution (*ν* = *µ*/*ρ* = 0.75 m^2^ s^−1^, *U* = 2.1 mm h^−1^, *L* =  ~ 1 µm) on the center *y*-position of 0.5 mm. The inertial forces are extremely smaller compared to viscous forces in these experiments, due to two primary reasons. Firstly, the xanthan solution used in the experiments has a high value of viscosity (e.g., ~ 0.45 Pa s at 40 °C at shear rate 10 s—1 for 1.0 wt% xanthan solution^21^). This extremely larger viscous force induced polymeric deposition on a substrate and bridging the gap, rather than homogenization of particles dispersion during water evaporation. Additionally, the gap length of the cells, is within the capillary length that leads to formation of a barrier by condensation of microparticles at the evaporative interface. This barrier, i.e. a skin layer enhances the increase of viscous force to make a nucleus for meniscus splitting. As time passed, the *Re* becomes smaller because of smaller *U* and larger *ν.* The spatio-temporal evaluation is now under consideration. Due that the drastic increase of the viscosity as the polymer concentration increases ~ 1 wt%^21^, time course changes of the viscosity of the fluid on the evaporative interface would be strongly related to the *Re* value.

**Figure 3:**
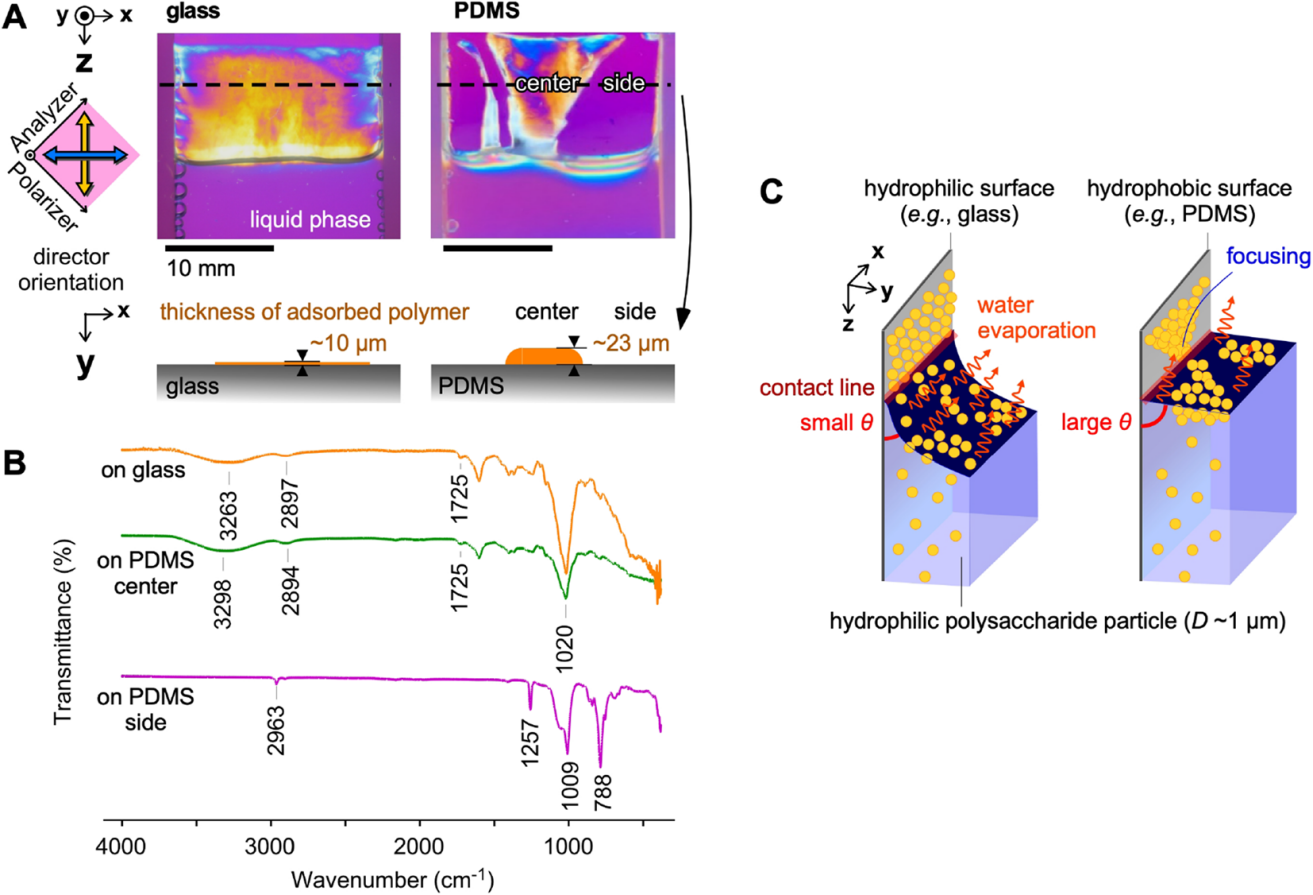
Evaluation of polysaccharide adsorption on glass substrates and PDMS substrates. (**A**) In-situ evaluation of adsorption under crossed-polarizers with a first-order retardation plate (λ = 530 nm) after 8-h drying at 60 °C. Inner dimensions of the cells: (*X*-width, *Y*-thickness, *Z*-depth) = (20 mm, 2 mm, ~ 20 mm). Initial polymer concentration: 1.0 wt%. (**B**) IR spectrum at given area on dotted lines. (**C**) Hypothesis of adsorption of polymeric particles on hydrophilic/hydrophobic surfaces by evaporative interface.

To clarify the effect of substrate wettability on meniscus splitting, the polymeric deposition during drying on a hydrophilic non-modified glass surface is compared with that on a hydrophobic polydimethylsiloxane (PDMS) surface (Fig. 3). In both the cases, nucleation for vertical membrane formation is observed around the center of the cell width. However, unlike the polymer adsorbed on the glass surface, that on the PDMS surface shows clearer focusing adsorption (Fig. 3A and SI Figs. S5 and S6). The thickness of the adsorbed polymer on the PDMS surface is 23 ± 2 µm, much larger than that on the glass substrate (10 ± 2 µm). In case of hydrophilic surface such as glass, it showed low contact angle of polymer solution drop due to a high surface energy. This led to a good adhesion of polymer on the surface through a concave meniscus. As a result, the polymer would widely adhere on the surface as a thin film on the *XZ*-plane. In contrast, hydrophobic surface such as PDMS, it showed high contact angle due to a low surface energy. The force of cohesion between the polymer/polymer overcomes the force of adhesion between polymer/surface. This causes a poor adhesion of polymer on the hydrophobic surface. The polymer flocculated in the *YZ*-plane to increase the interaction between polymer/polymer and to reduce the interaction between polymer/surface simultaneously. Thus, the polymer easily deposited as a thicker film in the *Y*-direction on hydrophobic surface. Furthermore, the Fourier-transform infrared (FT-IR) spectra at the given positions clearly differ, as shown in Fig. 3B. First, the area on the glass surface and the center area on the PDMS surface have characteristic peaks attributed to XG at 3280 cm^−1^ for alcoholic O–H stretching, 2897 cm^−1^ for carboxy O–H stretching, 1725 cm^−1^ for C=O stretching, and 1020 cm^−1^ for alcoholic C–O stretching. In contrast, the side area on the PDMS surface shows no peaks originating from XG but has characteristic peaks originating from PDMS: 2963 cm^−1^ for C–H stretching in CH_3,_ 1257 cm^−1^ for CH_3_ symmetric bending in Si–CH_3_, 1009 cm^−1^ for stretching of the siloxane bridge Si–O–Si, and 788 cm^−1^ for CH_3_ rocking in Si–CH_3_. These obvious differences indicate that the XG particles are integrated with focusing on the PDMS surface.

Such focus in deposition might be attributed to the large contact angle which provides smaller area for water evaporation (Fig. 3C). Considering that both cases have a similar evaporation rate on the whole, the water between the PDMS substrates would evaporate from smaller area and the integrated microparticle on the interface should become thicker. This situation induces a growth of thicker flocculated particles on the evaporative interface. Therefore, compared with particles on the evaporative interface between the hydrophilic surfaces, those between the hydrophobic surfaces like PDMS do not have strong adsorption on the hydrophobic surfaces. This condition induces that flocculated particle assemblies grow more easily on the evaporative interface. The growth of such flocculated assemblies creates a focusing deposition on a hydrophobic surface. Thus, the hydrophobic PDMS surface allow the hydrophilic particles to flocculate on the evaporative interface and deposit with focusing.

**Figure 4:**
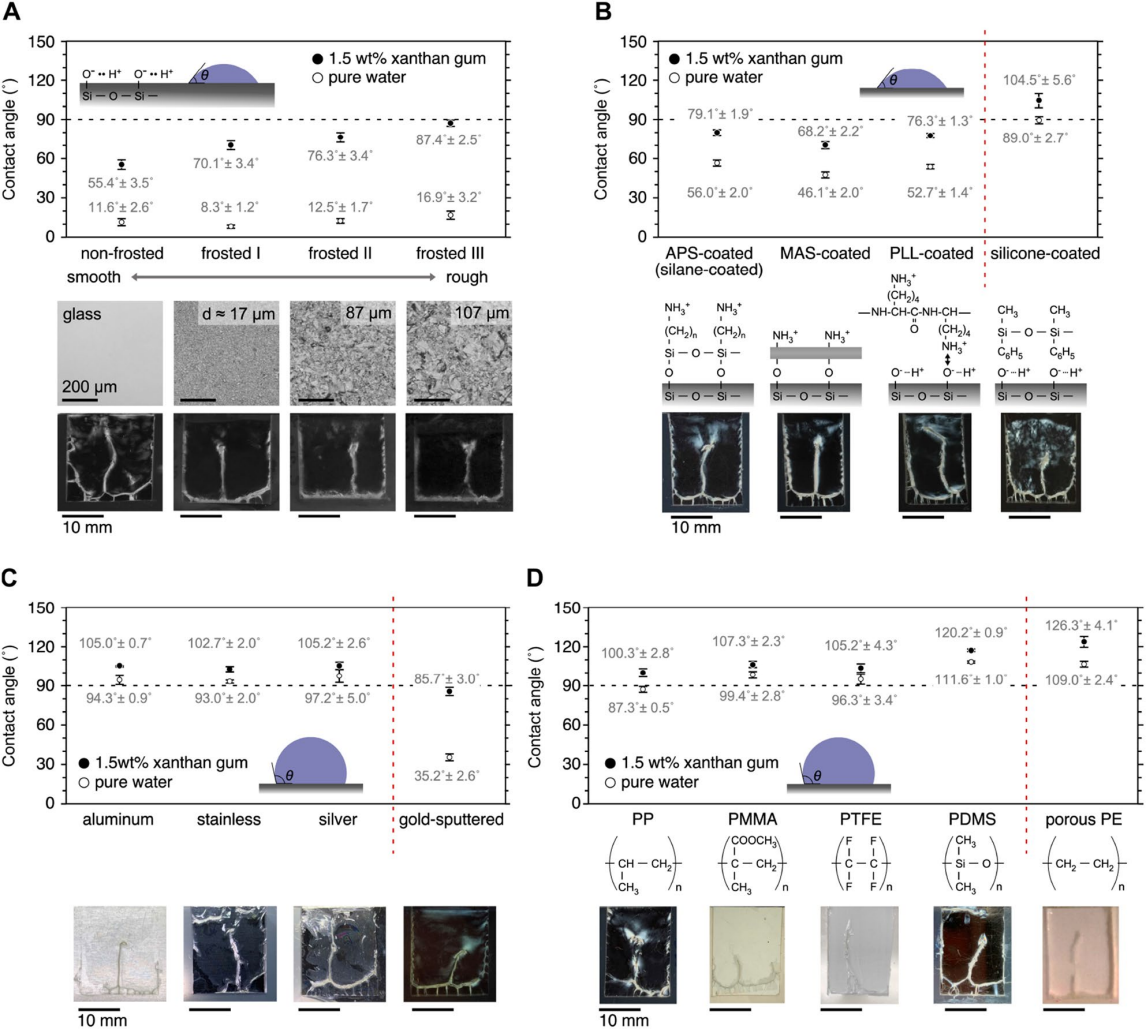
Macro-space partitioning on various kinds of substrates having hydrophilic/hydrophobic surfaces. Frosted glass (**A**), functional-molecules-modified glass (**B**), metals (**C**), and plastics (**D**). Contact angles on substrate for pure water and 1.5 wt% XG aqueous solution at 25 °C. Inner dimensions of the cell: (X-width, Y-thickness, Z-depth) = (15–20 mm, 1 mm, ~ 20 mm). Initial polymer concentration: 1.5 wt%. Drying temperature: 60 °C.

To apply specific deposits on different kinds of surfaces, meniscus splitting and vertical membrane formation are examined on frosted glasses (Fig. 4A). Surfaces with a wide range of roughness (*d* < 107 µm) allow the specific deposition of the polymer with vertical membrane formation because the flocculated particles on the evaporative interface are much larger than the micrometer-scale roughness. Therefore, the deposited polymers are adsorbed on the surfaces, forming bridging nuclei. On a frosted glass surface, a small increase in contact angle is obtained for water (*θ*_water_: 11° → 17°) with increasing surface roughness, while an extremely large increase (*θ*_XG_: 55° → 88°) is obtained for a 1.5 wt% XG solution. This means that the microparticles are easily integrated on the evaporative interface with a smaller interface area between the two substrates for bridging. To check membrane formation on substrates modified by functional molecules, glass substrates modified using aminopropyl triethoxysilane (APS-), Matsunami adhesive slide (MAS-), poly-l-lysine (PLL-), and silicone are used (46° < *θ*_water_ < 53°) (Fig. 4B). The negatively charged XG microparticles bridge the 1-mm gaps regardless of the surface charges (such as the positive charges of the APS-, MAS-, and PLL-modified glasses; negative charges of the non-modified glasses; and the neutral charge of the silicone surface). Thus, the charges have a somewhat small effect and the microparticle interaction during deposition on the air–water interface is significantly strong.

Deposition is also demonstrated on metals with hydrophobic surfaces (*θ*_water_ ≈ 95 ± 2°) (Fig. 4C). Similar to deposition on glass surfaces, metal surfaces like aluminum, stainless steel, and silver allowed meniscus splitting (SI Fig. S7). Such specific deposition still occurred on gold-sputtered surfaces with roughness (*θ*_water_ ≈ 86°). Thus, the partitioning phenomenon was observed on various metal surfaces. Similarly, deposition is studied on typical hydrophobic plastic surfaces, such as polypropylene (PP), polymethyl methacrylate (PMMA), polytetrafluoroethylene (PTFE), and PDMS (Fig. 4D). These hydrophobic substrates show large contact angles (85° < *θ*_water_ < 111°), yet still allow membrane formation. Membrane formation was also confirmed on a porous polyethylene substrate with an average pore size of 17 µm. On rough surfaces with poor wetting (*θ*_water_ ≈ 109°), the deposited microparticles also successfully bridged the 1-mm gap to form vertical membranes.

## Conclusion

Convective meniscus splitting was demonstrated by evaporating aqueous XG microparticle dispersions under controlled external conditions such as the direction of gravity, surface roughness, and surface wettability. Because the aqueous solution was highly viscous, the effect of direction of gravitational force was much smaller than that of the capillary force for this phenomenon. For further understanding, the correlations among the viscosity change, and the water evaporation rate would be revealed by discussing the Reynolds number in fluid dynamics in near future. We clarified that microparticles with diameters of ~ 1 µm were integrated through focusing to form the nucleus for bridging millimeter-scale gaps between two substrates. This demonstration would contribute the application of this phenomena in other kinds of polymers and smaller/larger size of the particle such as silica particles and microgel particles. The focusing of the microparticles occurred more strongly on hydrophobic surfaces like PDMS than on hydrophilic surfaces like glass, because the larger contact angle induced greater flocculation of microparticles on the evaporative interfaces. By using the evaporative air–LC interface, vertical membranes were successfully formed on various kinds of substrates, such as frosted glass, metals, and plastics, showing a wide range of water contact angles (11° < *θ*_water_ < 111°). These observations imply that the fingering phenomenon could be applied to space-partitioning and the formation of vertical polysaccharide membranes on many kinds of substrates. By introducing crosslinking points to the membrane, it could be used in mass separators and soft actuators with directional controllability^11,26,27^. In the future, we envision that this technique for splitting spaces with biopolymer membranes could be applied in microfluidic devices for preparation of a membrane with uniaxial orientation capable of controlling mass-separator.

## Methods

### Materials

XG (*M*_w_ = 4.7 × 10^6^ g mol^−1^) obtained from *Xanthomonas campestris*, neosoft XC, was purchased from Taiyo Kagaku Co. Japan. Glass substrates with a variety of surfaces were purchased from Matsunami Glass Ind. Ltd., Aluminum, stainless steel, copper (Kyuho Co., Japan), and silver (99.99%, Inushio Precious Metals Co. Ltd., Japan) substrates were used as-purchased. Plastic substrates made of PP resin (Harue Plastic Kogeisha, Japan), PMMA resin (Harue Plastic Kogeisha, Japan), PTFE (As One Co.), and porous polyethylene film with characteristic pore size of 17 µm (SUNMAP HP-5320, Nitto Denko, Co.) were used as-purchased. The PDMS-coated substrates were prepared by spin-coating a mixture (2 µL) of Sylgard 184 silicone elastomer base and Sylgard 184 silicone elastomer curing agent (Dow Corning Toray Co., Ltd.) on the glass substrates using a spin-coater (3000 rpm, 10 s). To fix the PDMS, it was placed in an oven at 110 °C for 2 h.

### Drying experiments

The drying condition for the aqueous solution was set according to our previous works^22^. After the XG was dissolved in pure water, the aqueous solutions were used within a few days. After removing air bubbles, the room-temperature aqueous solution was poured into a kind of one-side-open Hele-Shaw cell. The U-shaped cell is composed of two non-modified glass slides and glass spacers fixed with glue to prevent sample leakage. The gap distance is controlled by the thickness of the spacer. The cells were placed in an oven at constant temperature under atmospheric pressure with an air circulator (EYELA, DTC-41). Considering that the volume of the oven (192 mm × 270 mm × 192 mm, ~ 10 L) with the air circulator (effective exhaust velocity: ~ 40 L min^−1^) was much larger than that of the samples (< 1 mL), the relative humidity in the oven can be controlled by regulating the temperature. For the zero-gravity experiment, the gravity controller (Gravite, Space Bio-Laboratories, Co., Ltd.) was placed in an oven and used at 40 °C.

### Observations and characterizations

To verify the degree of orientation, the samples were photographed through linear crossed-polarizers (Supporting Information, Fig. S5)^11^. To clarify the orientation direction, a first-order retardation plate with λ = 530 nm was put into the light path between a polarizer and the sample. Microscopic observations were made using a microscope (BX51, Olympus) equipped with a CCD camera (DP80, Olympus)^11^. Fourier-transform infrared (FT-IR) spectra of the polymer adsorbed on a substrate were recorded on a spectrometer (Spectrum 100, Perkin Elmer) using a diamond-attenuated total-reflection (ATR) accessory.

## Supplementary Information

Supplementary Figures.
