# Supplementary material for: Convective meniscus splitting of polysaccharide microparticles on various surfaces
Source: Sci Rep. 2021 Jan 12;11:767. doi: 10.1038/s41598-020-80779-z (PMC7804455; doi:10.1038/s41598-020-80779-z)
Supplement: Supplementary file 1 — Supplementary Figures. [file 41598_2020_80779_MOESM1_ESM.pdf]

Supporting Information

Convective meniscus splitting of polysaccharide microparticles on various surfaces

Kosuke Okeyoshi*, Miki Yamashita, Kulisara Budpud, Gargi Joshi, and Tatsuo Kaneko*

Correspondence to: okeyoshi@jaist.ac.jp (KO); kaneko@jaist.ac.jp (TK)

Figure S1. Chemical structure and optical microscopic images of xanthan gum (XG) deposited on a substrate.

Figure S2. Standard Mollier diagram for estimation of the relation between temperature and humidity.

Figure S3. Schematic of direction of gravitational force and cell placement: parallel to the *Z*-axis, parallel to the *Y*-axis, and zero-gravity conditions.

Figure S4. Schematic of the experimental setup used for observations under cross-polarized light.

Figure S5. Time-course changes of water evaporation and polymer deposition from a cell comprising glass or PDMS substrates.

Figure S6. Contact angles on copper substrate for pure water and 1.5 wt% XG aqueous solution and drying test for macro-space partitioning.

A

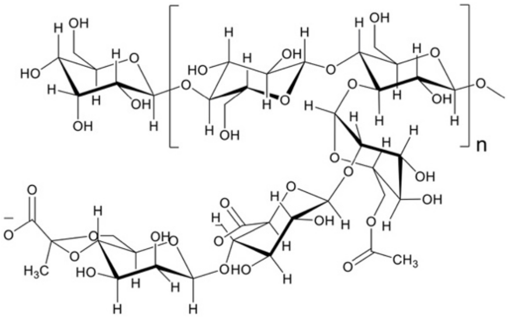

B

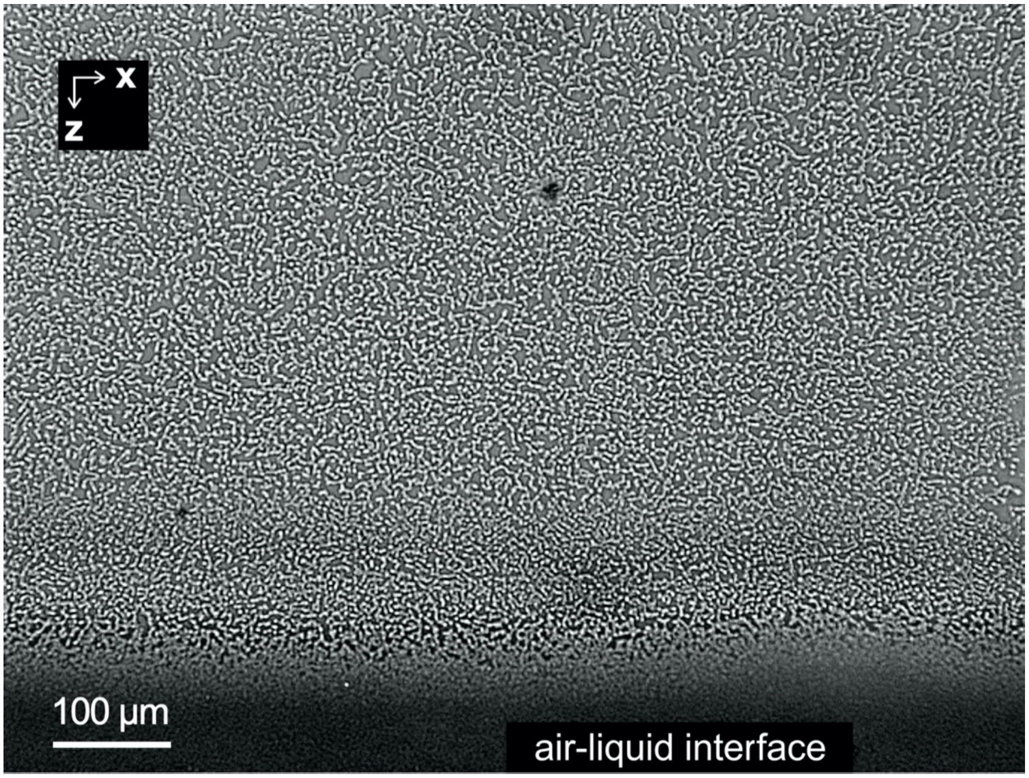

Figure S1. Chemical structure (A) and optical microscopic images of xanthan gum (XG) deposited on a substrate (B). Initial concentration of XG: 0.5 wt%.

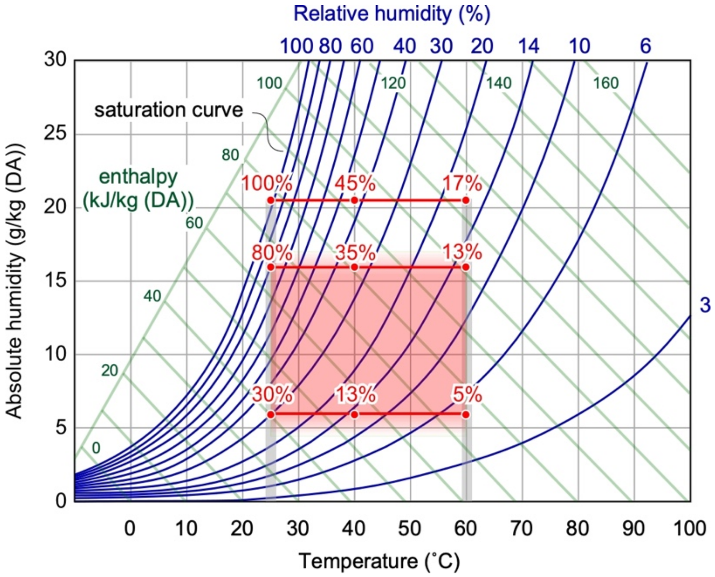

Figure S2. Standard Mollier diagram for estimation of the relation between temperature and humidity. DA = dry air.

The relative humidity in the area, Ishikawa, is within the range 30–80% RH in a typical year. Considering that the air in the oven is circulated from the room air, the relative humidity at 40 °C is estimated to be within the range 13–35% RH.

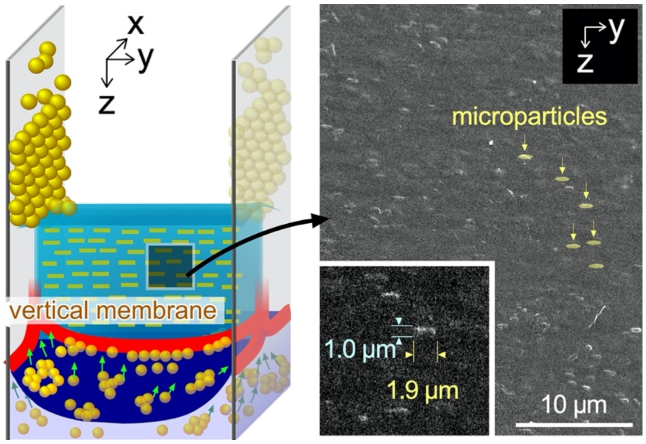

Figure S3. Scanning electron microscope image of vertical membrane surface. Inset: magnified view.

From the SEM image, the microparticles could be observed on the whole area of the vertical membrane surface. They seemingly have anisotropic shape with ~1 μm-width and ~2 μm-length.

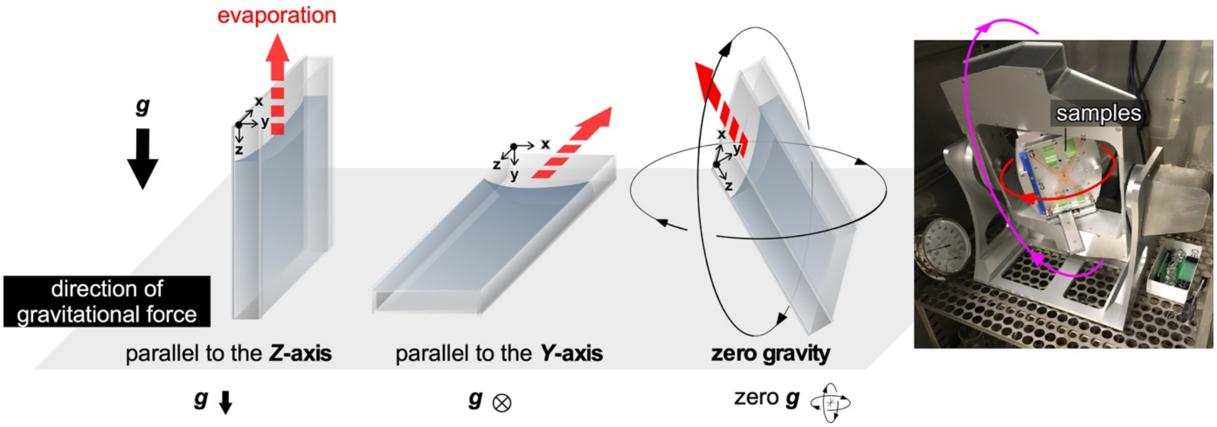

Figure S4. Schematic of direction of gravitational force and cell placement: parallel to the *Z*-axis, *Y*-axis, and zero-gravity. Photograph: setup for zero-gravity using a gravity controller (Gravite, Space Bio-Laboratories).

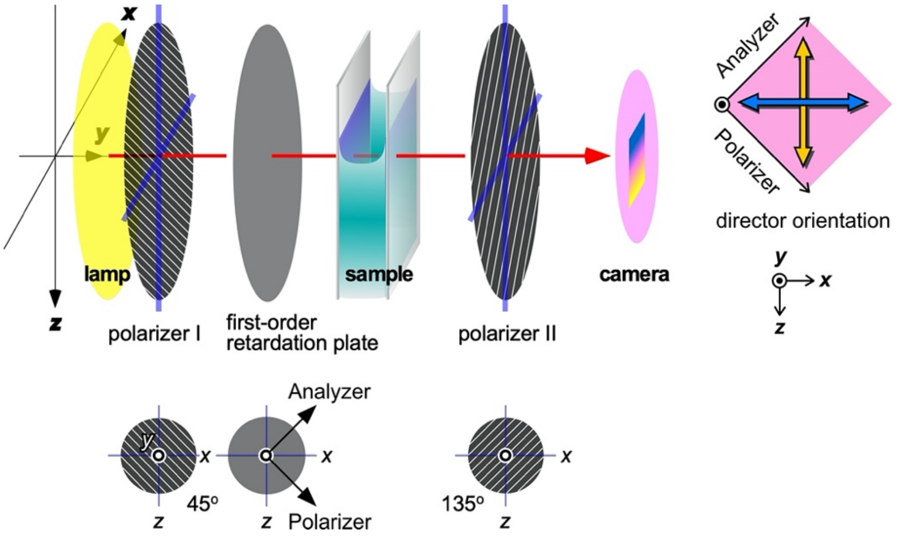

Figure S5. Schematic of the experimental setup used for observations under cross-polarized light. The polarizers were normally adjusted to 45° and 135°. A first-order retardation plate with λ = 530 nm was placed between the polarizer and the sample.

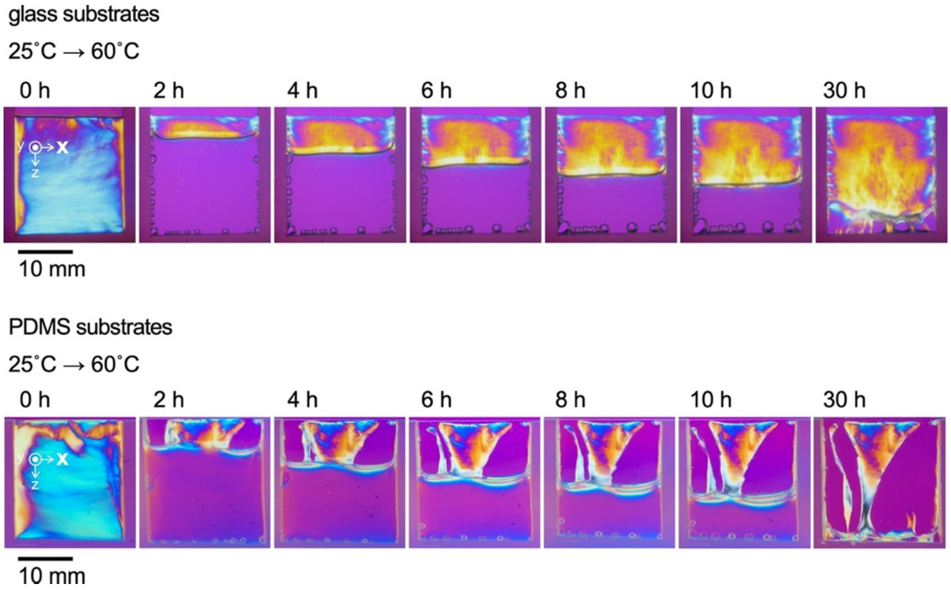

Figure S6. Time-course changes of water evaporation and polymer deposition from a cell composed of glass or PDMS substrates. Drying temperature: 60 °C. Inner dimensions of the cells: (*X*-width, *Y*-thickness, *Z*-depth) = (20 mm, 2 mm, ~20 mm).

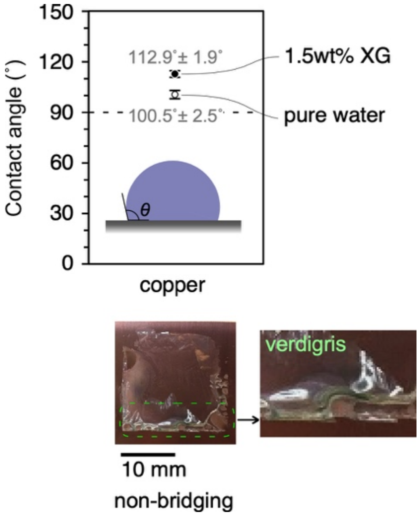

Figure S7. Contact angles on copper substrate for pure water and 1.5 wt% XG aqueous solution and drying test for macro-space partitioning. Inner dimensions of the cell: (*X*-width, *Y*-thickness, *Z*-depth) = (20 mm, 1 mm, ~20 mm). Initial polymer concentration: 1.5 wt%, Drying temperature: 60 °C.

Copper surface allowed neither splitting nor bridging deposition. A color change on the surface indicates that the water seemingly ionized the copper surface during drying. This ionized copper, *i.e.* verdigris, prevents specific polymer deposition to bridge the gap.
